# Online cognitive behaviour training for the prevention of postnatal depression in at-risk mothers: a randomised controlled trial protocol

**DOI:** 10.1186/1471-244X-13-265

**Published:** 2013-10-17

**Authors:** Bethany A Jones, Kathleen M Griffiths, Helen Christensen, David Ellwood, Kylie Bennett, Anthony Bennett

**Affiliations:** 1 Centre for Mental Health Research, The Australian National University, Building 63 Eggleston Road, Acton ACT 0200, Australia; 2 The Black Dog Institute, Prince of Wales Hospital, Hospital Road, Randwick, NSW 2031, Australia; 3 School of Medicine, Gold Coast campus, Griffith University, QLD 4222, Australia

**Keywords:** Postnatal depression, Perinatal depression, Postpartum depression, Cognitive behaviour therapy, E-health, Randomised controlled trial

## Abstract

**Background:**

Postnatal depression (PND) is the most common disorder of the puerperium with serious consequences for both mother and child if left untreated. While there are effective treatments, there are many barriers for new mothers needing to access them. Prevention strategies may offer a more acceptable means of addressing the problem. Internet interventions can help overcome some barriers to reducing the impact of PND. However, to date there are no published studies that investigate the efficacy of internet interventions for the prevention of PND.

**Methods/Design:**

The proposed study is a two-arm double blind randomised controlled trial. 175 participants will be recruited in the immediate postnatal period at an Australian community hospital. Women who meet inclusion criteria (internet access, email address, telephone number, over 18, live birth, fluent English) will complete the Edinburgh Postnatal Depression Scale (EPDS). Those with a score above 9 will undertake the Structured Clinical Interview for DSM Disorders (SCID). Those with a clinical diagnosis of depression, or a lifetime diagnosis of bipolar disorder or psychosis on the SCID will be excluded. Following completion of the baseline battery women will be randomised using a computer-generated algorithm to either the intervention or control condition. The intervention will consist of 5 modules of automated, interactive cognitive behaviour training (CB training), completed weekly with email reminders. The control will replicate the level of contact participants experience with the intervention, but the content will be of a general health nature. Participants will complete questionnaires immediately post-intervention (6 weeks) and 3-, 6- and 12 months follow-up. There will also be a second SCID delivered via telephone at 6 months. We hypothesise that relative to the control group, the intervention group will show a greater reduction in postnatal distress on the EPDS (primary outcome measure). We also hypothesise that the intervention group will demonstrate lower levels of anxiety and stress and higher levels of parenting confidence than the control group following intervention and/or follow-up.

**Discussion:**

The proposed study addresses a number of limitations of earlier trials.

**Trial registration:**

Australia and New Zealand Clinical Trials Registers, ACTRN12609001032246.

## Background

Postnatal depression (PND) is defined by the Diagnostic and Statistical Manual of Mental Disorders (DSM-IV) [1] as an episode of major depression commencing within the first 4 weeks postpartum. In practice, most researchers and clinicians broaden the onset period to include any time in the first 12 months postpartum. With a prevalence rate of approximately 13% [2], PND is the most common complication of the puerperium. Not only is PND relatively common, but it also has a profound effect on both mother and child. Women experience low mood, low satisfaction with parenting and feelings of guilt. In extreme cases they may attempt or complete suicide or infanticide. Studies of infants have shown that the effects of PND on the child, including poor attachment and cognitive deficits, persist well into childhood [3].

As with other depressive disorders, there are a number of effective treatments for PND including antidepressant medication and psychotherapy. However there are unique barriers to implementing treatments for PND. The first is that breastfeeding women may be unwilling to take antidepressant medications for fear of the effects on their baby [4,5]. Secondly, new mothers are often time-poor and this can be a major barrier to undertaking cognitive behaviour therapy (CBT) [6]. Women with a new baby are typically unable to schedule lengthy sessions with a health professional. Finally, new mothers may be particularly susceptible to perceived and self-stigma. Stigma may serve as a barrier to help seeking for any type of depression. However, new mothers with depression may be subject to the additional concern that they will be perceived as a “bad mother” as a result of their condition. As a consequence they may refuse to seek help, refuse treatment when diagnosed, and even refuse to accept a diagnosis of depression [7]. Because of these unique barriers, prevention of PND, rather than treatment, may be a particularly appropriate course to pursue.

There has been substantial research into the prevention of PND. A recent review [8,9] found that prevention programs delivered postnatally only were more beneficial than those that included an antenatal component. Also, interventions delivered individually were more effective than those delivered to groups. Some prevention studies have focussed on models of care, where the delivery of service has been the focus, rather than the content of the intervention. Some of these models include postnatal layperson follow-up and home-based care. These strategies have not been particularly effective [8,9]. This is not surprising, given that the content of many of these interventions (support and/ or education about PND) was not necessarily selected on the basis of demonstrated efficacy in other contexts. Other studies that have used individual or group therapy based on Cognitive Behaviour Therapy (CBT) or Interpersonal Therapy (IPT), which are of known efficacy in *treating* postnatal depression, have been more successful in reducing symptoms and preventing the development of PND [10-14]. This type of intervention however, is expensive. Prevention involves delivering interventions to large numbers of women. To provide individual or group therapy to such numbers is costly and unlikely to constitute a feasible strategy for preventing PND.

### Rationale for the proposed study

The Internet offers a potential means for addressing the barriers associated with the delivery of conventional prevention programs to women in the post natal period. Such programs can be accessed online without cost. They do not require time consuming, and inconveniently scheduled, trips to face-to-face sessions. Rather, a mother can access the intervention at times and for periods which are short enough to fit into their baby’s variable and demanding schedule. They can do as much or as little at a time that suits them. Moreover, the anonymity of accessing an Internet intervention may overcome the stigma associated with seeking help for the subclinical symptoms of PND [15]. It may be argued that there are inequities inherent in an Internet-delivered intervention; however, this inequity of access is no greater, and arguably less than with traditional interventions. In many jurisdictions, those disadvantaged women who do not have access to the Internet are also unlikely to be able to afford face-to-face interventions. Additionally, within the geographical area of this trial, around 90-95% of the target population has Internet access [16].

There is now convincing evidence that Internet-based programs based on CBT are effective for treating depression [17]. There is also evidence of their efficacy in preventing depression [18-20]. To date there have been no published studies of the efficacy of internet interventions for treating or preventing PND. However, we have undertaken a pilot survey of young mothers (n = 34, unpublished) to examine the acceptability of an automated CB Training web-based program for PND. The findings suggest that such a program would be acceptable to this group.

Therefore, the current study seeks to evaluate the efficacy for preventing PND of an Internet intervention program, MoodGYM. This automated Cognitive Behaviour Training (CB Training) web-based program has been demonstrated in randomised controlled trials to reduce depressive symptoms in members of the community at risk of depressive disorder [20] and to prevent new cases of depression and anxiety in young people [19]. Since a recent meta-analysis concluded that the most effective preventions have been delivered solely in the postnatal period [8,9], the proposed intervention will be undertaken in this period.

Previous studies of the efficacy or effectiveness of PND prevention programs have been characterised by a number of weaknesses. One key limitation is that they have failed to employ an adequate control condition often using instead a usual care scenario e.g. [21-23], or wait list control e.g. [24] neither of which explicitly controls for the effects of the increased contact with staff. The current study will employ an attention control condition that matches the level of contact provided to the intervention group. Another limitation of previous PND prevention studies is that they have failed to exclude women with a current diagnosis of PND. Our proposed study will exclude women with an existing disorder enabling the true prevention effect of the intervention to be examined in the absence of confounding from treatment effects.

## Methods/Design

### Design

A 2 (Condition) X 4 (Time) double blind, randomised controlled design is planned. Participants will be randomly allocated to either the online intervention or an online control condition. Outcomes will be assessed at baseline, post-intervention (6 weeks), 3 months, 6 months, and 12 months post-randomisation.

### Participants

Participants will be recruited in the maternity (postnatal) ward and birthing centre of The Canberra Hospital in Canberra, Australia. Women will be eligible for the trial if they have internet access, an email address, a telephone number, are over 18, have had a live birth, can read and write English, and score over 9 on the Edinburgh Postnatal Depression Scale. Women who are currently receiving formal psychological treatment will be excluded from the trial. Potential participants will be asked if they are seeing a health professional for a mental health condition and if so, the nature of their treatment. If the treatment they are receiving is any of the ‘talking’ therapies, they will be excluded. Those receiving exclusively pharmacological interventions will be not be excluded. Women with a current diagnosis of a depressive disorder, or a history of mania or psychosis will also be excluded (based on SCID modules A and B – Mood Episodes and Psychotic Symptoms). Women who endorse self-harm items will also be excluded.

### Measures

The primary outcome will be postnatal distress. The secondary outcomes will be the severity of depressive and anxiety symptomatology, parenting confidence and diagnostic status. These will be measured as follows:

1. *Postnatal distress* will be measured online using the Edinburgh Postnatal Depression Scale (EPDS) [25]. The most widely-used measure of postnatal depression and anxiety, this instrument contains 10 items and takes around 5 minutes to complete. Answers are scored between 0 and 3, and summed for a total score (range 0 to 30) with higher scores indicating higher levels of postnatal distress. The validity of the EPDS has been established within the Australian population [26], for online administration [27] and for use in the immediate postpartum period to predict PND outcomes e.g. [28,29]. The cut-off used in this study (over 9) is that recommended by Matthey *et al.*[30] for probable minor depression.

Secondary outcomes

2. *Depressive and anxiety symptomatology* will be measured online using the Depression, Anxiety and Stress Scale (DASS) [31], a 21 item self-report questionnaire designed to measure the severity of symptoms. This self-report measure has adequate psychometric characteristics [32] and has been previously used in this population [33].

3. *Parenting confidence* will be assessed using the 15-item self-report Karitane Parenting Confidence Scale (KPCS) [34]. Although the KPCS is a new measure, it shows good test-retest reliability and internal consistency. The scale was developed and tested in Australia, making this a particularly appropriate instrument for the current study. The KPCS will be delivered via the Internet.

4. *Depression Status* will be evaluated using the Structured Clinical Interview for DSM Disorders (SCID) Module A (Mood Episodes). The SCID is a structured interview for current and past diagnoses of DSM-IV disorders. There have been a number of reliability studies yielding an acceptable range of Kappa values for the diagnosis of major depression (from 0.61 [35] to 0.93 [36]). At baseline this interview will be delivered face-to-face; however, at 6-months follow-up it will completed via telephone [37].

5. *Risk factors* to be assessed by self-report include: health of infant, sex of infant, infant feeding status and satisfaction, infant feeding and sleep patterns, major life events, and difficulty of pregnancy and delivery. Each of these will be assessed with a single item. For example, “How satisfied are you with your birth experience with this baby” has a 5-point Likert Scale from ‘Very satisfied’ to ‘Very dissatisfied’. We will also assess level of maternal support using the Maternal Social Support Scale, and personal history of depression through the SCID. All risk factor items will be administered as part of an online battery, with the exception of the SCID.

6. Cognitive behaviour therapy literacy and stigma will be assessed using an online delivery of the CBT literacy scale and the Depression Stigma Scale (DSS) [38,39]. The CBT literacy scale has previously been successfully used to assess change in CBT knowledge following a MoodGYM intervention [20] and the DSS has adequate reliability and validity.

7. *Socioeconomic factors* will be assessed online, including self-reported age, marital status, education level, occupation, income, and parity.

### Conditions

#### The intervention condition: MoodGYM

MoodGYM is an online, interactive program delivering cognitive behaviour training. The program consists of five modules which take between 20 and 40 minutes to complete. However, participants will be able to choose to spend as much or as little time as they wish in a given session. Module 1 (Feelings) will help participants to identify negative thinking patterns. Module 2 (Thoughts) will assist them to identify inaccurate and biased ways of thinking. Module 3 (Unwarping) will present methods for changing their negative and biased habitual thinking patterns. Module 4 (De-stressing) will focus on stress management and relaxation techniques. Finally, in module 5 (Relationships and Problem Solving), the participant will be provided with techniques for coping with difficult relationships. It will also introduce the concept of simple problem solving. Each module includes some work that is completed immediately and some that users return to and expand (the users’ ‘Workbook’). Some examples of these ‘Workbook’ exercises are identifying unhelpful thought patterns and using specific tools to contest them. Users can also schedule pleasant activities, and other ‘homework’ exercises.

Participants will complete one module per week. They will be required to complete each module before they can proceed to the next. Participants will be sent an automated email each week to inform them when the next module is available. Participants can complete each module at their own pace. However, a reminder will be sent if the participant fails to log in to the website within four days of receiving the initial notification that the next module is available.

#### Control condition

Participants will receive a series of five, weekly emails directing them to online modules containing general wellbeing information (HealthWatch website as described in the WellBeing Trial protocol [40]). Participants will complete questionnaires and interactive modules on information pertaining to health issues. The content of the modules will be as follows. Module 1: Diet and Nutrition. Module 2: Back pain. Module 3: Oral Health. Module 4: Calcium and Vitamin D. Module 5: Environmental Health. The modules also include items that ask participants about how these health issues impact on mental health.

### Sample size

Based on power calculations, an estimated minimum of 112 participants are required. Assuming a correlation of .5 between baseline and endpoint outcome measures, a 20% dropout, and an effect size of 0.43 [10], this sample size will have the power at 80% to detect differences at endpoint of .54 standard deviations (alpha = 0.05, non-directional test). Approximately 3000 babies are born per year at The Canberra Hospital. 20% of their mothers will score over 9 on the EPDS immediately after birth [33,41], yielding a potential pool of 600 women per year. Of these women, roughly 4% [42] will be clinically depressed and therefore excluded, leaving a potential pool of 576. Based on rates of recruitment from community samples, we anticipate that 25% of these potential participants will agree to participate in the study, yielding a sample of 144 women per year. Allowing for a further 10-20% unexpected drop-out after recruitment and before randomisation this should be sufficient to reach the target sample size of 112 participants.

### Randomisation

Participants will be randomised to one of the two website conditions using a computer-generated algorithm that is replicable and validated. The start value for the seed will be determined from the current server time and will be recorded for replication. Randomisation will not be stratified and will occur automatically at the completion of the baseline survey by taking the next allocation of the generated schedule.

### Blinding

The automated randomisation of participants after baseline completion will enable the enrolling researcher to remain blind to participant condition over the course of the trial. Those who administer the 6-month SCID interview, and who analyse the data will also remain blind to condition. The active nature of the control condition may also allow us to blind participants to condition. Participants will be informed that the purpose of the study is to investigate the efficacy of two online programs to prevent postnatal depression. However, they will not be explicitly advised of the content or intention of either site. This minor deception was designed to yield a double-blind trial of the psychological intervention. To this end, the Human Research Ethics Committees of ACT Health and the Australian National University have approved the procedure. To assess the success of blinding, participants will asked to indicate whether they were assigned to the condition that we anticipated to be more successful or the condition we expected to be less successful. This assessment will be performed immediately post-intervention.

### Analysis

The outcome variables will be analysed on an intent-to-treat (ITT) basis using a linear mixed models analysis, a procedure which is able to handle missing data [43] with timepoints as a within groups factor and intervention condition as a between groups factor. A significance level of 0.05 will be used for all outcome variables. We plan to analyse contrasts between intervention and control groups at post intervention, 3-months, 6-months and 12-months follow-up.

### Procedure

Ethical approval has been obtained from ACT Health Human Research Ethics Committee and The Australian National University Human Research Ethics Committee.

Women will be approached by a member of the research team while in hospital who will explain the study and offer information sheets and consent forms where appropriate. Those who consent to participate will be administered an inclusion/ exclusion criteria questionnaire by the enrolling researcher. Women who satisfy these criteria will be assessed using the Edinburgh Postnatal Depression Scale (EPDS), self-administered on a notebook computer. Women who score under 9 will not be eligible. Those who score 9 or over will then be assessed for depressive disorders, bipolar disorder or history of psychosis using a face-to-face administration of the Structured Clinical Interview for DSM Disorders (SCID). Women who have a diagnosis will be excluded from the study, as will those who scored under 9 on the EPDS.

**Figure 1:**
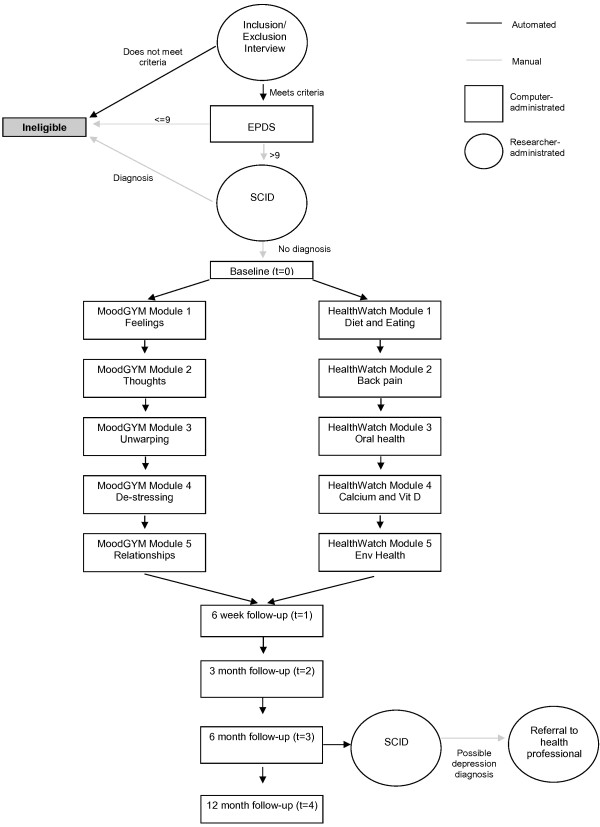
Study flowchart.

Participants will then complete the baseline questionnaires online using a notebook computer, after which they will be randomised either to the intervention or control condition (Figure 1). After 1 week, participants will receive an email informing them that the first online module is available. They will then complete either the MoodGYM or HealthWatch modules weekly for 5 weeks.

All participants will be invited to complete online follow-up questionnaires immediately post-intervention (6 weeks) and at 3 months, 6 months, and 12 months post-randomisation (Table 1). The 6 month SCID will be completed via telephone. Participants who develop a clinical diagnosis in the course of the trial, or contact trial staff with concerns about their wellbeing, will be referred to an appropriate health professional.

**Table 1:** Schedule of questionnaires

**Instrument/ question**	**Baseline**	**6 wks**	**3 m**	**6 m**	**12 m**
Inclusion criteria questionnaire	✓	**-**	**-**	**-**	**-**
Edinburgh Postnatal Depression Scale (EPDS)	✓	✓	✓	✓	✓
Depression Anxiety and Stress Scale (DASS)	✓	✓	✓	✓	✓
Karitane Parenting Confidence Scale (KPCS)	✓	✓	✓	✓	✓
Structured Clinical Interview for DSM Disorders (SCID)	✓	-	-	✓	-
Health of infant	✓	✓	✓	✓	✓
Infant feeding status and satisfaction	✓	✓	✓	✓	✓
Level of support (MSSS)	✓	✓	✓	✓	✓
Major life events	✓	**-**	**-**	✓	✓
PND/ Depression literacy	✓	**-**	**-**	**-**	✓
Have you sought help for PND	-	✓	**-**	✓	✓
Personal details and contact information	✓	**-**	**-**	**-**	**-**
SES: age, marital status, education, occupation, income	✓	**-**	**-**	**-**	**-**
Family history of depression	✓	**-**	**-**	**-**	**-**
Personal history of depression	✓	**-**	**-**	**-**	**-**
Personal history of abuse	✓	**-**	**-**	**-**	**-**
Parity	✓	**-**	**-**	**-**	**-**
Stressfulness of pregnancy	✓	**-**	**-**	**-**	**-**
Difficulty of delivery	✓	**-**	**-**	**-**	**-**
Sex of infant	✓	**-**	**-**	**-**	**-**

## Discussion

The aim of this trial is to assess the effectiveness of online CB Training for the primary prevention of postnatal depression in an at-risk sample. To our knowledge there are no published studies investigating the use of an online intervention to prevent PND.

We hypothesise that the MoodGYM prevention group will show a greater reduction in postnatal distress as measured by the EPDS. We also hypothesise that they will demonstrate lower levels of anxiety and stress and higher levels of parenting confidence than the control group.

The current study addresses many of the shortcomings of earlier trials. Many trials targeted “at-risk” women, but failed to screen out women with a current disorder. By contrast, the current study excludes women with a diagnosis of depression and thus is a true prevention trial not contaminated by treatment effects which inflate the effect size. Most previous PND prevention trials have used interventions that been previously untested either as *treatments* for PND or as treatments or preventive interventions for depression in general. Therefore the significance of a failure of the intervention to prevent PND is difficult to interpret. MoodGYM is an intervention of demonstrated efficacy both as a treatment, and as prevention for depression in a range of non-PND populations. Similarly, many of the studies using CBT incorporate additional interventions in the prevention program. Again, this complicates the interpretation of the study findings. We propose to use CB Training by itself rather than as part of a multi-component intervention. This will allow us to draw conclusions about the utility of Cognitive Behavioural strategies specifically for PND prevention.

The major limitation of the study is its small geographic coverage. For pragmatic reasons, the study will not, at this stage, be conducted in more than one site. The recruited sample may therefore not be representative of the broader population. Canberra’s population is better educated and of higher socio-economic status than the average for Australia. Further, since there have been no previous studies of online CB Training interventions for this population the recruitment rate is difficult to predict with certainty. A further potential limitation of the intervention is that it is not tailored to PND specifically. On the other hand, it possible that a generic intervention could have a normalising effect on symptoms.

If effective, MoodGYM could be conveniently implemented in postnatal contexts across Australia. It is currently widely accessible with over 450,000 registrants since 2006. There would also be value in investigating the efficacy of MoodGYM as a universal preventive intervention (delivered to women regardless of their level of depressive symptoms). The architecture of MoodGYM enables translations to be undertaken and trials to be run distally in other countries. MoodGYM has already been translated into Norwegian, Dutch and Chinese, and the arrangements for translations into other languages are in progress. This will facilitate future cross-cultural comparisons of the utility of MoodGYM for prevention of PND.

## Competing interests

KG is the Director of e-hub’s self-help Web Services, and KG and HC are developers of MoodGYM. They do not benefit financially from MoodGYM. There are no other competing interests.

## Authors’ contributions

BJ conceived the study, participated in its design and drafted the paper for publication. KMG, HC, DE, KB and AB participated in the study design and edited the paper. All authors read and approved the final manuscript.

## Pre-publication history

The pre-publication history for this paper can be accessed here:

http://www.biomedcentral.com/1471-244X/13/265/prepub
